# Evaluation of Diet Supplementation with Wheat Grass Juice on Growth Performance, Body Composition and Blood Biochemical Profile of Carp (*Cyprinus carpio* L.)

**DOI:** 10.3390/ani11092589

**Published:** 2021-09-03

**Authors:** Cristian-Alin Barbacariu, Marian Burducea, Lenuta Dîrvariu, Eugen Oprea, Andrei-Cristian Lupu, Gabriel-Ciprian Teliban, Alina Laura Agapie, Vasile Stoleru, Andrei Lobiuc

**Affiliations:** 1 Research and Development Station for Aquaculture and Aquatic Ecology, “Alexandru Ioan Cuza” University, Soseaua Iasi-Ciurea, km 5, 700718 Iasi, Romania; alin.barbacariu@uaic.ro (C.-A.B.); dirvariu.lenuta@gmail.com (L.D.); eugen.oprea@uaic.ro (E.O.); 2 Veterinary Medicine Department, “Ion Ionescu de la Brad” University of Life Sciences, 3 M. Sadoveanu, 700440 Iasi, Romania; lupuandrei@protonmail.com; 3 Horticulture Department, “Ion Ionescu de la Brad” University of Life Sciences, 3 M. Sadoveanu, 700440 Iasi, Romania; gabrielteliban@uaiasi.ro (G.-C.T.); vstoleru@uaiasi.ro (V.S.); 4 Agricultural Research and Development Station Lovrin, 200, 307250 Lovrin, Romania; alinamartinig@yahoo.com; 5 Human Health and Development Department, “Stefan Cel Mare” University, Universitatii Street, 720229 Suceava, Romania; andrei.lobiuc@usm.ro

**Keywords:** *Cyprinus carpio* L., phytogenics, wheat grass juice, growth indices, body composition, blood biochemical profile

## Abstract

**Simple Summary:**

Phytogenics are feed additives of plant origin, that gained considerable attention due to the wide range of benefits for farmers and animals, as well as for the environment. Phytogenics can be used to stimulate growth, strengthen the immune system, promote intestinal flora, or reduce intestinal inflammation in fish. In this study, the effect of feed supplementation with wheatgrass juice as a phytogenic on the growth, body composition and blood biochemical profile in carp was tested. The results showed that wheat grass juice produces positive effects on growth, contributing to higher final biomass while reducing the fat content of meat. At the same time, the content of albumin, globulin, total protein, and calcium in the blood increased in wheat grass juice feeds. This study opens the premises for the use of wheat grass juice as a feed additive for carp.

**Abstract:**

Wheat grass juice (WGJ) is an extract of young wheat plantlets (*Triticum aetivum* L.) used worldwide for its health related properties. In this study, the following feeds containing WGJ were tested on common carp (*Cyprinus carpio* L.): Control (C), WGJ1% (V1), WGJ2% (V2) and WGJ4% (V3) *w*/*w*. Fish with an average initial weight of 102 g/individual were grown in a recirculating aquaculture system. The results showed that WGJ had stimulatory effects on growth performance. Accordingly, final body weight increased by 11% at V1, 39% at V2 and 23% at V3, while other indices (feed conversion ratio, specific growth rate, relative growth rate, protein efficiency ratio, and condition factor) were unaffected. Body composition analyses revealed a significant decrease in fat content at V2 and a significant increase in collagen and ash at the same variant, while the protein content was unmodified. Regarding the blood profile, significant increases in the content of albumin, globulin, total protein, and calcium were recorded in the variants with WGJ. The positive results of WGJ on carp can be attributed to its biochemical composition, which is rich in chlorophyll (4.71 mg mL^−1^), total phenols (164 µg mL^−1^ gallic acid equivalents), and high antioxidant activity (67% inhibition of DPPH 2,2-diphenyl-1-picrylhydrazyl). The results suggest WGJ can be used as a promising feed additive for common carp.

## 1. Introduction

Common carp (*Cyprinus carpio* L.) is one of the most cultivated fish species, representing 7.7% of world production in 2018 (4189.5 thousand tons) [1]. In Romania, common carp is the most cultivated species, with 4365.45 tons produced in 2018, representing 35% of the total (12,298.45 tons) [2]. The popularity of common carp in Romania follows the consumers’ perception as being a healthy source of meat, while the main way of cultivation is extensive or semi-intensive in polyculture with other cyprinids in ponds, with locally produced feed. In addition, carp aquaculture in ponds also brings ecosystem services, many of the farms being integrated into protected natural areas.

In order to meet the high market demand, farmers are required to increase the density of fish stocks, causing negative consequences such as oxidative stress, decreased immunity and ultimately decreased yield [3]. A solution for this problem is the use of additives that improve fish growth, enhance disease resistance and reduce the effects of oxidative stress due to high densities or variations in physical-chemical parameters of water [4]. Natural additives bring more benefits and may improve animal welfare, protect the environment, and increase food safety for people. Numerous studies have been conducted on the use of additives of plant origin, proving positive effects on growth, immunity, digestion, intestinal microbiota, reproductive performance [5,6]. Plant origin additives are called phytogenics and can include essential oils, plant extracts, plant juices, powders [7,8] etc. The effects of phytogenics on fish depend mainly on their chemical composition, for example the main effect of essential oils administration in feeds is immunity boost due to their antimicrobial properties [9,10,11], while aqueous plant extracts may improve body weight [12]. The use of phytogenics have been reported to improve blood parameters in fish such as plasma protein, total plasma lipid, Hb, Ht, AST, ALT, and uric acid concentrations [13]. In addition to the nutritional and therapeutic effects, the use of phytogenics adds value to the production system by being environmentally friendly and having a low cost of production compared to synthetic additives [14].

Wheat grass juice (WGJ) is an extract from young wheatgrass plants (10 days) of *Triticum aestivum* Linn. and is considered to have multiple therapeutic properties such as antioxidant, immunological, cardioprotective, blood sugar modulator etc. [15,16,17,18]. The biochemical composition of WGJ is complex containing mainly chlorophyll. Other components are: minerals (Ca, P, Mg, K, Zn, B), vitamins (A, C, E, B complex), enzymes (amylases, cytochrome oxidase, proteases, transhydrogenase, superoxide dismutase), amino acids (aspartic acid, glutamic acid, arginine, alanine, serine), phenolic acids (ferulic, gallic, syringic, p-coumaric, caffeic) and flavonoids (apigenin, luteolin and quercetin) [19].

In a previous study, we demonstrated that the addition of WGJ to carp feed (1%, 2%, and 4% *w*/*w*) had positive effects on growth and on the activity of oxidative stress enzymes (SOD, CAT, and GPX) and lipid peroxidation in muscle and liver tissues [20]. The present study aims to evaluation of the effects of wheat grass juice on the growth performance, body composition and biochemical profile of the blood in order to recommend an optimal concentration for carp feeding.

## 2. Materials and Methods

### 2.1. Fish Trial

The experiment was carried out within the Research and Development Station for Aquaculture and Aquatic Ecology “Alexandru Ioan Cuza” University, Iasi, Romania. *Cyprinus carpio* L., with an initial weight of 102 g/individual were grown in an indoor recirculation aquaculture system (RAS). The RAS is equipped with fiberglass growth tanks (0.75 m^3^/tank), mechanical drum type filter, UV filter, biological filter, pump supply system, and sensors for monitoring the physical and chemical parameters. Natural light was supplemented with fluorescent tubes for 10 h per day, while salinity was 0.1 mg/L. Water parameters are presented in Table 1. Temperature and content of dissolved oxygen were measured with Hach HQ30d portable oxygen meter (Hach Company, Loveland, CO, USA), pH and conductivity with a Hach HQ11d (Hach Company) portable multiparameter, while ammonia, nitrites, nitrates, and phosphates were assessed with Hanna Iris HI801 Spectrophotometer and Hanna reagent kits (Hanna Instruments, Salaj, Romania).

### 2.2. Wheat Grass Juice Extraction

Wheatgrass juice was obtained according to the method described in Dumitru et al. (2018) [20]. Wheat seeds were germinated in the dark for 3 days in 1 L black plastic pots, filled with commercial soil (Florisol Product S.R.L., Dorohoi, Romania). After the germination, plants were grown in natural light, and watering was performed twice a day with 50 mL/pot. After 10 days of growth, the plants reached the optimal harvest height (15 cm). The plants were harvested by cutting the plants at a distance of 2 cm from the soil. Wheatgrass juice was obtained by cold pressing.

The following feeds containing feed + wheat grass juice (WGJ) were tested: Control (C–100% feed), WGJ1% (V1–WGJ 1% + FEED 99%), WGJ2%, (V2–WGJ 2% + FEED 98%), and WGJ4% (V3–WGJ 4% + FEED 96%) *w*/*w*. Wheat grass juice was prepared daily and was included in feed using a sprayer for a uniform distribution. The feeds were left for 30 min to dry and then administered to the fish. Fish were weighed weekly in order to adjust the amount of feed according to manufacturer indications (https://www.aller-aqua.com/species/warm-freshwater-species/carp (accessed on 1 May 2021). In the first week 5.65 kg feed per 100 kg fish biomass was used and 4.52 kg feed per 100 kg of fish in the following weeks. For each feed 30 fish with uniform initial weight were selected. The survival rate was 100%. The feed used was Aller classic with a pellet size of 3 mm. The proximate composition of feed was performed with DA 7250 NIR Analyzer, (Perten Instruments, Hagersten, Sweden) and the results are presented in Table 2.

### 2.3. Chemical Analysis of Wheat Grass Juice

After extraction, wheat grass juice was refrigerated until analysis of chlorophyll a, chlorophyll b, carotenoids, total phenols, total flavonoids, and the antioxidant activity.

Chlorophyll was extracted in acetone (80%) and the optical density was read at 470, 646 and 663 nm. Pigments content was calculated using the equations described in Wellburn (1994) [21].

Total phenolics, total flavonoids and antioxidant activity were determined according to the methods described by Lobiuc et al. (2017) [22].

Briefly, the total phenolic content was quantified using Folin–Ciocalteu method, and results were expressed based on a gallic acid calibration curve as mg gallic acid equivalent (GAE)/mL. Total flavonoid content was assessed using AlCl_3_ reagent and results were expressed based on a quercetin calibration curve as mg quercetin equivalent (QE)/mL. Antioxidant activity was measured according to the DPPH assay, the results being expressed as % inhibition of DPPH.

### 2.4. Fish Growth Indices

Weekly, fish length and weight (w) were measured (*n* = 15).

IBW—initial body weight (g);
FBW—final body weight (g);
WG—weight gain (g) = FBW − IBW;
FCR—feed conversion ratio (g/g) = Feed intake (g)/WG;
RGR—relative growth rate (g/g day^−1^) = WG/days of experiment/IBW;
SGR—specific growth rate (% day^−1^) = (ln FBW − ln IBW)/days of experiment × 100;
PER—protein efficiency ratio = WG/total protein;
CF—condition factor = FBW/body length^3^ × 100.

### 2.5. Body Composition

Proximate body composition parameters were calculated using DA 7250 NIR Analyzer, (Perten Instruments).

### 2.6. Blood Parameters

The fish were anesthetized with clove oil (2%), and blood was sampled by heart puncture. The samples were analyzed with the MNCHIP Pointcare V2 Analyzer. The biochemical parameters were: ALB—albumin, TP—total protein, GLO—globulin, A/G—albumin/globulin, Ca^2+^—calcium, GLU—glucose, BUN—blood urea nitrogen, AMY—amylase, CHOL—cholesterol, ALT—alanine aminotransferase, TBIL—total bilirubin, ALP—alkaline phosphatase, CRE—creatinine, CK—creatine kinase.

### 2.7. Statistical Analysis

The data were statistically processed by ANOVA followed by Tukey Test (*p* ˂ 0.05) using the SPSS software version 21 (IBM Corp, Armonk, NY, USA). The results were reported as means ± standard errors.

## 3. Results

### 3.1. Yield and Biochemical Composition of Wheat Grass Juice

The yield and chemical composition of WGJ is presented in Table 3. The yield of WGJ obtained by cold pressing of young wheat plants (10 days) is high, considering the fact that from 100 g of wheat grass, 78.5 g of juice were obtained. Of the total chlorophyll pigment content of WGJ 49.6% is represented by chlorophyll a, 42.8% chlorophyll b, and 7.5% carotenoids. WGJ also contains phenols and, in particular, flavonoids, which impart with high antioxidant activity (67%).

### 3.2. Fish Growth Performance

**Figure 1:**
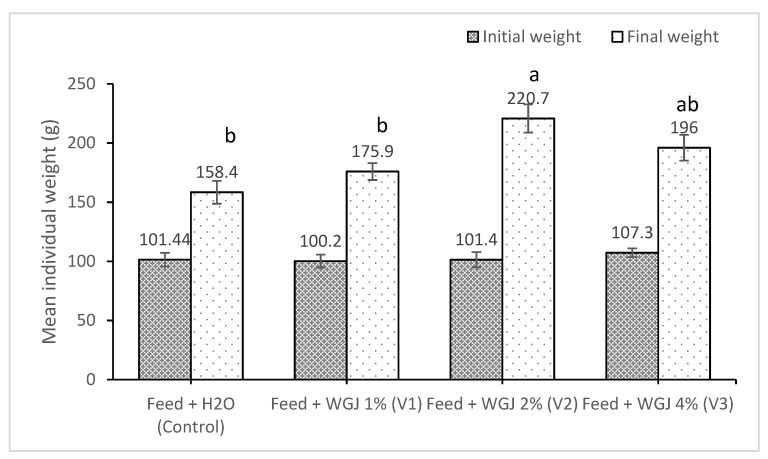
Initial body weight and final body weight of common carp feed four diets with different inclusion levels of wheat grass juice (WGJ). Results are means ± standard error, small letters—statistically significant differences, Tukey test (*p* < 0.05).

The effect of WGJ on carp body weight is presented in Figure 1. The results indicate that carp feed diets supplemented with WGJ increased the final body weight in all WGJ feeds (V1–11%, V2–39%, and V3–23%) compared with control, with V2 significantly different from the control (*p* = 0.0007).

**Figure 2:**
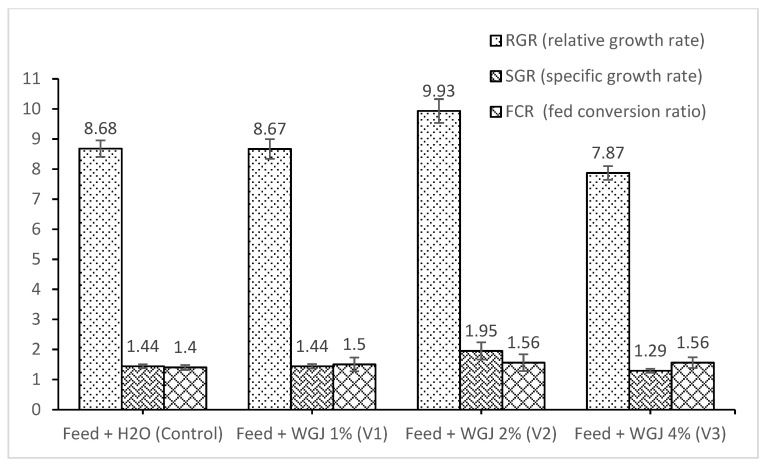
Performance of common carp feed four diets with different inclusion levels of wheat grass juice (WGJ): RGR—relative growth rate, SGR—specific growth rate, and FCR—feed conversion ratio. Results are means ± standard error.

RGR, SGR and FCR are shown in Figure 2. RGR and SGR were highest in V2 proving the beneficial effect of WGJ, while FCR was lowest in the Control variant, however the differences were statistically insignificant (*p* > 0.05). 

Initial and final body length are presented in Table 4. The largest statistically significant increases of the final body length compared to the control variant was recorded in variant V2 (7%). Weight gain was two times higher in V2 compared to control variant. No statistically significant differences were recorded for PER and CF, however CF results correlate with those from WG, which reflects a positive effect of WGJ on carp growth (Table 4). 

### 3.3. Body Composition

Body composition is presented in Table 5. Except for protein, all parameters were significantly influenced (*p* ˂ 0.05) by WGJ. The lowest fat content was recorded at V2, 2.5 times lower than the Control. The collagen and ash content were significantly higher at V2.

### 3.4. Blood Parameters

The results of the biochemical profile of the blood are presented Table 6. Total proteins, albumin and globulin were significantly increased in WGJ treated fish, more precisely ALB + 15% at V1, TP + 6% at V3 and GLO + 25% at V3. Calcium levels increased significantly also in fish feed with WGJ (+12%, at V2). Glucose levels were not altered by WGJ, while BUN increased significantly at V2 by 26%. Cholesterol and amylase levels decreased significantly in this study in the 2% WGJ-feed variant. WGJ did not significantly (*p* > 0.05) influenced the enzymes ALT and ALP, as well as TBIL, CRE and C/K.

## 4. Discussion

The use of phytogenics to combat the stress caused by high stocking densities or to improve growth performance has become an alternative method to chemical additives. Researchers are making efforts to identify new sources of phytogenics with positive effects on many different fish species [23,24,25]. The identification of effective plant additives for inclusion in feed is particularly important in the context of a global trend to replace ingredients of marine origin with some of plant origin, and also because of the fact that feed is the main production cost in the aquaculture sector [26,27,28].

Herein, wheat grass juice (WGJ) was used as an additive in the feeding of common carp. The effects on growth performance, feeding efficiency, body composition and blood biochemical profile were tested. Regarding the biochemical composition of WGJ, chlorophyll, phenolic and flavonoid contents and the antioxidant activity were determined. Chlorophyll content was relatively high (4.71 mg mL^−1^) compared with results on WGJ published by Özköse et al. (2016) [29] (25.4–27.9 mg 100 mL^−1^), and lower compared with barley juice (6.62 mg g^−1^) [30]. Chlorophyll is the major chemical constituent of cereal grass juice (wheat, barley, oats) representing up to 70% of the total, and for this reason WGJ is called “green blood”. Chlorophyll is responsible for the green color of plants, being one of the most abundant phytochemicals in nature. Chlorophyll has been studied for about 100 years for its therapeutic properties due to its resemblance to the chemical structure of hemoglobin [31,32]. Recently, chlorophyll derivatives have been reported to have systemic activities and to regulate oxidative stress and the expression of genes responsible for preventing cancer [33]. Another class of bioactive compounds found in WGJ are total phenols and total flavonoids (164 µg mL^−1^ and 289 µg mL^−1^, respectively), although the values are lower than in [29] (342 mg L^−1^ and 598 mg L^−1^, respectively) the antioxidant activity was high (67% DPPH% inhibition). Variation in biochemical composition of plant extracts is normal and is dependent on plant species and cultivar, cultivation conditions, extraction techniques and methods of investigation [34] These compounds are widely used in medicine due to their health related benefits, such as prevention or reduction of heart disease and diabetes, as well as antibacterial, antiviral, anti-inflammatory, and anti-allergenic properties [35,36].

Regarding the fish growth in this study, final weight increased up to 39% and body length increased up to 7% in common carp feed with 2% WGJ. SGR and RGR, PER, and CF were also higher than control in the same variant. The positive results are in line with the general trend presented by the literature regarding the use of phytogenics, however some variations may occur depending on the plant species from which the active principle is extracted and the cultured fish species [37]. For instance, in this study FCR varied between 1.4 (Control) and 1.56 (V2 and V3), while other studies reported a decrease of FCR after phytogenics administration [38]. FCR is a very important indicator because it reflects how much feed is used to obtain 1 kg of fish. Feed producers generally optimize the feed recipes so that the FCR is as low as possible, however variations may occur depending on the type of aquaculture, water quality, temperatures, etc. [39,40]. The use of phytogenics in common carp may contribute to the decrease of FCR, as shown by Mocanu et al. (2018) [38] who managed a decrease of FCR from 1.6 to 1.2% using plant extracts (garlic and seabuckthorn) in a proportion of 4%.

Body composition analysis revealed a considerable decrease in fat content, from 2.53% at control to 0.9% at V2 (2% WGJ). The fat content is one of the most important feature in the marketing of carp and is significantly influenced by the growing conditions. Due to the fact that the content of fat varies greatly (from 2.7% to 17.6%) and a content over 15% can adversely affect the taste of the meat, some countries only allow the marketing of carp with a content below 10% [41,42,43]. Moreover, the collagen and ash content were significantly higher in the same variant, which demonstrates the positive effects of WGJ administration in feeding of common carp. Considering the fact that in some studies the ash and protein content increased and in others there were no changes due to the application of phytoadditives, we can conclude that their influence on body composition is dependent on the type of additive and the species studied [44]. 

Under experimental conditions, blood analysis can provide valuable information about fish physiology and health status [45]. For example, Latif et al. (2021) [46] established that dietary supplementation with black seed in rohu has a nephroprotective effect by decreasing creatinine and urea levels, and hepatoprotective by decreasing the activity of some enzymes (ALP, AST and ALT). In this study, WGJ did not significantly (*p* > 0.05) influence the enzymes ALT and ALP, as well as CRE, C/K, and GLU level, while AMY level increased, thus we can consider that WGJ has a low hepatoprotective activity compared to other literature reports. Moreover, BUN level, which is a product of protein metabolism and an indicator of kidney and gills health, has significantly increased which may suggest kidney damage. On the other hand, in this study, variants with WGJ had a higher content of total protein, albumin, globulin, and Ca, which may suggest an immunological role of WGJ [47]. Our results are in agreement with Bao et al. (2019) [47], who found an increase in the content of total protein, albumin and globulin in carp exposed to *Aeromonas hydrophila* infection after dietary administration of *Gingko biloba* leaf extract. Another important effect obtained in this study is the significant decrease of cholesterol and amylase levels at 2% WGJ-feed variant. Cholesterol plays a multiple physiological role, being a precursor of all steroid hormones, being a component of cell membranes. The literature presents different effects of phytoadditives on cholesterol levels. Thus, Yang and Chen (2003) [48] showed that the administration of a garlic extract in carp feed increases the cholesterol, while Kesbic (2018) [49] found that cholesterol, triglyceride and ALP levels decreased, while ALT and total proteins increased in carp as a result of juniper oil additive. The positive effects on maintaining normal histoarchitecture and metabolic enzymes levels are considered to be due to bioactive compounds in phytoadditives with antioxidant role [46]. The presence of bioactive compounds may justify both the antioxidant effect of WGJ, which was confirmed by increased activity of antioxidant enzymes in muscle and liver tissue [20], and the positive effects on growth, body composition and blood profile recorded in this study.

## 5. Conclusions

Wheat grass juice (WGJ) was tested as a feed additive for common carp. WGJ contain bioactive compounds such as chlorophyll pigments and phenolic compounds, with high antioxidant activity. The results in this study indicate that the addition of WGJin carp feed improves growth performance (particularly at 2% feed supplementation), by increasing the final body weight with Over 1/3. Growth indices (FCR, SGR, RGR, PER and CF) were not significantly influenced by WGJ. Meat quality was improved by a significant decrease in fat content and a significant increase in collagen and ash, while protein content was unmodified. In addition, WGJ addition improves blood markers, such as the content of blood albumin, globulin, total protein, and calcium and by lowering cholesterol. Wheat grass juice can be considered a promising feed additive for common carp. Further studies need to be done in order to find the optimal inclusion method (such as spraying) for different production scales.

## Figures and Tables

**Table 1:** Average values of the water parameters in RAS.

Parameters	Week 1	Week 2	Week 3	Week 4	Week 5	Week 6	Week 7
Temperature (°C)	22	22	22	22	22	22	22
pH (pH units)	7.9	7.8	7.8	7.9	8	7.9	7.9
Dissolved oxygen (mg/L)	9.79	9.01	8.78	8.58	8.90	9.12	9.40
Conductivity (µS/cm^3^)	1271	1240	1222	1305	1277	1295	1283
Nitrates (NO^3−^)(mg/L)	39.6	37.1	44	35.2	26.4	30.8	35.2
Nitrites (NO^−^) (mg/L)	0.10	0.11	0.13	BD	BD	0.06	BD
Ammonia (NH3^+^) (mg/L)	BD	BD	BD	BD	BD	BD	BD
Phosphates (mg/L)	0.01	0.01	0.006	0.006	0.01	0.002	0.006

BD—below detection limit.

**Table 2:** Feed composition (Aller classic, pellet size 3 mm).

Parameters	Feed + H_2_O (Control)	Feed + WGJ 1% (V1)	Feed + WGJ 2% (V2)	Feed + WGJ 4% (V3)	*p*-Value
Moisture	9.36 ± 0.01	8.95 ± 0.03	9.11 ± 0.03	9.96 ± 0.03	0.00
Protein	28.12 ± 0.11	27.54 ± 0.17	27.6 ± 0.16	26.97 ± 0.18	0.00
Fat	4 ± 0	4.17 ± 0.03	4.02 ± 0	3.93 ± 0.03	0.00
Ash	9.02 ± 0.03	9.46 ± 0.22	9.63 ± 0.24	9.39 ± 0.14	0.18
Phosphorus	1.06 ± 0.02	1.09 ± 0.01	1.07 ± 0.02	1.02 ± 0.01	0.04
Fiber	2.47 ± 0.04	2.45 ± 0.01	2.47 ± 0.04	2.72 ± 0.04	0.00

Values are means (*n* = 3) ± standard error, WGJ—wheat grass juice.

**Table 3:** Biochemical composition of wheat grass juice.

Parameters	Values
Fresh yield (%)	78.5 ± 2.01
Chlorophyll a (mg mL^−1^)	2.34 ± 0.03
Chlorophyll b (mg mL^−1^)	2.01 ± 0.03
Carotenoids (mg mL^−1^)	0.35 ± 0.01
Total chlorophyll (mg mL^−1^)	4.71 ± 0.06
Total flavonoids (QE µg mL^−1^)	289.24 ± 4.72
Total phenols (GAE µg mL^−1^)	164.81 ± 2.99
Antioxidant activity (DPPH% inhibition)	67.78 ± 4.08

Values are means (*n* = 3) ± standard error.

**Table 4:** Performance of common carp feed four diets with different inclusion levels of wheat grass juice (WGJ).

Indices	Feed + H_2_O (Control)	Feed + WGJ 1% (V1)	Feed + WGJ 2% (V2)	Feed + WGJ 4% (V3)	*p*-Value
IBL (cm)	17.63 ± 0.28	17.52 ± 0.33	17.45 ± 0.35	17.35 ± 0.37	0.22
FBL (cm)	19.98 ± 0.3 ^b^	20.26 ± 0.24 ^b^	21.34 ± 0.27 ^a^	20.96 ± 0.27 ^ab^	0.00
WG (g)	56.96 ± 3.88 ^b^	75.7 ± 1.64 ^b^	119.3 ± 5.45 ^a^	88.7 ± 7.14 ^ab^	0.00
PER	0.54 ± 0.02	0.57 ± 0.07	0.6 ± 0.15	0.46 ± 0.06	0.77
CF	1.99 ± 0.05	2 ± 0.03	2.12 ± 0.04	2.05 ± 0.03	0.14

IBL—initial body length, FBL—final body length, WG—weight gain, PER—protein efficiency ratio, CF—condition factor. Results are means ± standard error, small letters—statistically significant differences, Tukey test (*p* < 0.05).

**Table 5:** Body composition of common carp feed experimental diets with different inclusion levels of wheat grass juice (WGJ).

Indices	Feed + H_2_O (Control)	Feed + WGJ 1% (V1)	Feed + WGJ 2% (V2)	Feed + WGJ 4% (V3)	*p*-Value
Fat (%)	2.53 ± 0.03 ^b^	2.97 ± 0.03 ^a^	0.9 ± 0.06 ^d^	1.27 ± 0.03 ^c^	0.00
Protein (%)	15.3 ± 0.26	15.17 ± 0.03	15.03 ± 0.24	15.4 ± 0.29	0.70
Collagen (%)	1.3 ± 0.15 ^ab^	1.13 ± 0.03 ^b^	1.7 ± 0.1 ^a^	1.27 ± 0.09 ^ab^	0.02
Ash (%)	1.9 ± 0.12 ^ab^	1.07 ± 0.03 ^b^	2.2 ± 0.15 ^a^	1.77 ± 0.37 ^ab^	0.02

Results are means ± standard error, small letters—statistically significant differences, Tukey test (*p* < 0.05).

**Table 6:** Blood biochemical profile of common carp feed four experimental diets with different inclusion levels of wheat grass juice (WGJ).

Parameter	Feed + H_2_O (Control)	Feed + WGJ 1% (V1)	Feed + WGJ 2% (V2)	Feed + WGJ 4% (V3)	*p*-Value
ALB (g/dL)	1.5 ± 0.06 ^b^	1.73 ± 0.03 ^a^	1.57 ± 0.06 ^ab^	1.7 ± 0.07 ^ab^	0.02
TP (g/dL)	3.4 ± 0.06 ^b^	3.53 ± 0.03a ^b^	3.57 ± 0.03 ^ab^	3.63 ± 0.06 ^a^	0.04
GLO (g/dL)	1.67 ± 0.09 ^b^	1.87 ± 0.09 ^ab^	1.93 ± 0.12 ^ab^	2.1 ± 0.03 ^a^	0.02
A/G	0.87 ± 0.03	0.93 ± 0.03	0.83 ± 0.03	0.83 ± 0.03	0.19
Ca (mg/dL)	7.5 ± 0.2 ^b^	8.3 ± 0.2 ^ab^	8.47 ± 0.07 ^a^	8.1 ± 0.07 ^ab^	0.04
GLU (mg/dL)	95 ± 1	95.33 ± 2.96	92.33 ± 0.88	97.33 ± 2.73	0.40
BUN (mg/dL)	2.98 ± 0.09 ^b^	2.35 ± 0.04 ^c^	3.78 ± 0.43 ^a^	3.02 ± 0.31 ^b^	0.00
AMY (U/L)	64.33 ± 1.2 ^a^	71.67 ± 1.2 ^a^	46.33 ± 6.84 ^b^	64 ± 7.75 ^a^	0.00
CHOL (mg/dL)	214.67 ± 0.33 ^a^	218.33 ± 2.03 ^a^	190.33 ± 7.81 ^b^	213.67 ± 10.07 ^a^	0.00
ALT (U/L)	45.67 ± 3.18	35.33 ± 2.91	38 ± 1.67	38.67 ± 1.2	0.07
TBIL (mg/dL)	0.19 ± 0.01	0.23 ± 0.01	0.23 ± 0.01	0.24 ± 0.01	0.10
ALP (U/L)	34 ± 1	31.67 ± 1.67	32.33 ± 1.67	30.67 ± 0.67	0.07
CRE (mg/dL)	0.69 ± 0.04	0.68 ± 0.01	0.66 ± 0.04	0.62 ± 0.06	0.63
CK (U/L)	1745 ± 81.07	1962 ± 136.07	1834 ± 87.44	1976.67 ± 83.67	0.28

Results are means ± standard error, small letters—statistically significant differences, Tukey test (*p* < 0.05).

## Data Availability

All data used in this study are presented in this article.
